# S10-2 U.S. CDC’s Active People Health Nation: Michigan’s Story for Promoting Physical Activity in Local Communities

**DOI:** 10.1093/eurpub/ckad133.049

**Published:** 2023-09-11

**Authors:** Xining Yang, Tsu-Yin Wu

**Affiliations:** Eastern Michigan University, Center for Health Disparities Innovation and Studies (CHDIS); Eastern Michigan University, Center for Health Disparities Innovation and Studies (CHDIS)

## Abstract

**Purpose:**

Being physically active is one of the most important ways people of all ages and abilities can improve their health. Survey data show that 76 percent of Asian Americans in Michigan USA do not meet the federal physical activity minimum guidelines. Guided by Active People, Healthy Nation (APHN), a national initiative to help 27 million Americans become more physically active, the Eastern Michigan University (EMU) Racial and Ethnic Approaches to Community Health (REACH) initiative promotes physical activity through local partnerships in geographic areas where the priority population (Asian Americans) lives.

**Methods:**

Working with the state department of transportation, local municipalities, public schools, faith-based organizations, local businesses, and community health partners, EMU REACH used the following strategies to increase physical activity: 1) implement policies and activities to connect pedestrian, bicycle, or transit transportation networks (i.e., activity-friendly routes) to everyday destinations. 2) launch safe routes to school programs to increase walking and bicycling to and from school and 3) promote equitable park programs and policies that make it safe and easy for residents to be physically active. The team adopted the action planning guide and assessment modules in the Active Communities Tool (ACT) to facilitate cross-sector collaborations in developing an action plan to improve the community built environments that promote physical activity.

**Results:**

The EMU CHDIS has successfully reached 24,510 people impacted by new/improved policies and plans in 2018-22. In the process, our environment scan showed that people living in underserved areas and from racial and ethnic minority groups are less likely to have access to these places due to historical land use, housing, and transportation policies. Based on the results from project implementation, it is recommended that creating or modifying streets, parks, and trails to connect activity-friendly routes to everyday destinations and offer access to safe places for physical activity for people of all ages and abilities can promote active lifestyles and provide physical-active environment.

**Conclusion:**

While celebrating wins through the APHN initiative in Michigan, we advocate for new supportive policies and engage with more community members to raise awareness about the new resources to maintain the active momentum.

